# Kawasaki disease following Rocky Mountain spotted fever: a case report

**DOI:** 10.4076/1752-1947-3-7320

**Published:** 2009-07-06

**Authors:** Aswine K Bal, Steven W Kairys

**Affiliations:** 1 Division of Pediatric Infectious Diseases, K. Hovnanian Children's Hospital at Jersey Shore University Medical Center, Corlies Avenue, Neptune, New Jersey 07754-0379, USA; 2 Division of Pediatrics and Adolescent Medicine, K. Hovnanian Children's Hospital at Jersey Shore University Medical Center, Corlies Avenue, Neptune, New Jersey 07754-0379, USA

## Abstract

**Introduction:**

Kawasaki disease is an idiopathic acute systemic vasculitis of childhood. Although it simulates the clinical features of many infectious diseases, an infectious etiology has not been established. This is the first reported case of Kawasaki disease following Rocky Mountain spotted fever.

**Case presentation:**

We report the case of a 4-year-old girl who presented with fever and petechial rash. Serology confirmed Rocky Mountain spotted fever. While being treated with intravenous doxycycline, she developed swelling of her hands and feet. She had the clinical features of Kawasaki disease which resolved after therapy with intravenous immune globulin (IVIG) and aspirin.

**Conclusion:**

This case report suggests that Kawasaki disease can occur concurrently or immediately after a rickettsial illness such as Rocky Mountain spotted fever, hypothesizing an antigen-driven immune response to a rickettsial antigen.

## Introduction

Kawasaki disease (KD) is an acute febrile systemic vasculitis of childhood and is the leading cause of acquired heart disease in children in the United States and Japan [1]. Despite the availability of effective therapy to prevent cardiac complications, KD still remains an etiologic enigma. Although many epidemiologic and laboratory studies have looked at the linkage of Kawasaki disease to an infectious etiology, none of these associations has been proven [2]. We describe a patient who was diagnosed with Rocky Mountain spotted fever (RMSF) and treated successfully. During her acute illness, she developed clinical features of Kawasaki disease, which resolved with intravenous immune globulin (IVIG) and aspirin therapy.

## Case presentation

A 4-year-old Caucasian girl was admitted to hospital with fever, rash and bilateral conjunctival injection without eye discharge. Four days before hospitalization, she developed fever and a diffuse, non-itchy, erythematous rash on her extremities and trunk. On that evening, her mother noticed a dog tick attached to her scalp. She was seen in the emergency room and the tick was removed. She developed vomiting and non-bloody diarrhea on the following day. The next day, she developed redness of both eyes without any discharge. She also complained of pain in both her knees and her ankles while walking. She had no significant past medical illness. She had received all vaccinations appropriate for her age including second dose MMR (measles, mumps, rubella) vaccine. The family had a dog in the house. There was no history of any travel. None of her family members were ill during or preceding her illness.

**Figure 1:**
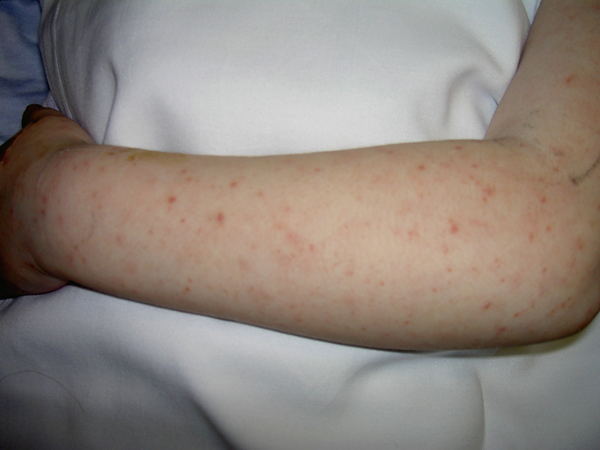
**Rash on upper extremity**.

**Figure 2:**
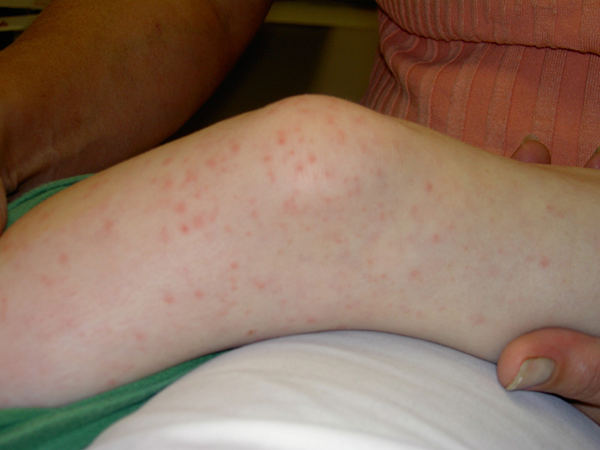
**Rash on lower extremity**.

On admission, she had fever of 39.4 °C. Her heart rate was 110/minute and her blood pressure was 82/58 mmHg. She had bilateral bulbar conjunctival injection without discharge. Her pharynx was slightly injected without any significant tonsillar enlargement, strawberry tongue or erythema of her lips. Her neck was supple without cervical lymphadenitis. Examination of her cardiac and respiratory systems was normal. Her abdomen was soft with her liver palpable 1 cm below the right costal margin. She had both a blanching macular and a petechial rash on her trunk and extremities including palms and soles (Figures 1 and 2). Her laboratory results showed WBC 3500/mm^3^ (5500-15,500/mm^3^), 48% polymorphonuclear leucocytes, 1% bandform, 45% lymphocytes, 6% monocytes, hemoglobin 8.9 gm/dL (11.5-12.5 gm/dl), hematocrit 24.7% (35-40%), platelet count 51,000/mm^3^ (150,000-350,000/mm^3^), ESR 52 mm/hour (4-20 mm/hour), sodium 130 mmol/L (133-146 mmol/L), alanine aminotransferase 178 IU/L (10-40 IU/L), aspartate aminotransferase 178 IU/L (1-50 IU/L), gamma glutamyl transferase (GGT) 94 IU/L (0-23 IU/L), direct bilirubin 1.3 gm/dL (0.3-1.2 mg/dL), normal urine analysis. A throat culture and ASO titers were normal. The cerebrospinal fluid (CSF) analysis showed protein of 31 mg/dL (5-40 mg.dL), glucose of 45 mg/dL (40-80 mg/dL), red blood cell count of 1/mm^3^, white blood cell count of 37/mm^3^ (0-7 WBC/mm^3^), 85% polymorphonuclear cells and 15% mononuclear cells. Blood culture, urine culture and serology for RMSF, leptospirosis and ehrlichiosis were dispatched. Her chest radiogram was normal.

She was treated empirically on intravenous doxycycline for presumptive diagnosis of RMSF along with ceftriaxone for possible bacterial sepsis. Over the next 48 hours, she became less febrile and her rash improved. But she had some residual macular rash without any worsening or new rash. RMSF IgM antibody was equivocal and IgG antibody was negative by ELISA. The blood, cerebrospinal fluid and urine culture remained negative and ceftriaxone was discontinued. After 1 week of her illness, her electrocardiogram (ECG) was normal and she had a small posterior/inferior pericardial effusion on echocardiogram.

On the ninth day of illness, she became very irritable. Her lips appeared erythematous. She developed redness and non-pitting swelling of her hands and feet. She received IGIV at a dose of 2 g/kg along with aspirin at a dose of 100 mg/kg/day for Kawasaki disease. Over the next 12 hours, she became afebrile. Swelling of her hands and feet resolved in 48 hours and the macular rash resolved completely within 72 hours following IGIV therapy. Her pancytopenia resolved in 48 hours after IGIV therapy. She was discharged after 1 week of hospitalization. Her clinical course, hematologic parameters and echocardiogram have been normal on follow-up. Her serology for Epstein–Barr virus, group A Streptococcus, and leptospira infection was normal. CSF for viral culture and enterovirus polymerase chain reaction (PCR) were negative. She received high-dose aspirin (80 mg/kg/day) until she remained afebrile for 96 hours following which she was placed on low-dose aspirin (4 mg/kg/day) for 6 weeks. Her ECG and echocardiogram were normal at 3 and 6 weeks after the onset of her illness.

## RMSF antibody results

On the 6th day of illness: (ELISA) IgM-equivocal, IgG-negative. On the 12th day of illness: (ELISA) IgM-positive, IgG-equivocal. In the 5th week after onset of illness: (ELISA) IgM-positive, IgG-positive; (IFA) IgM: >1:1024, IgG 1:512.

An RMSF antibody titer (IFA) less than 1:64 is considered negative. RMSF antibody by ELISA was done at ARUP laboratory (Salt Lake City, UT, USA) and IFA was done at Nicholas Institute (San Juan Capistrano, CA, USA).

## Discussion

Our patient presented with a history of tick bite, fever, petechial rash, gastrointestinal symptoms, pancytopenia, hyponatremia and elevated liver enzymes. Her RMSF antibody showed positive IgM antibody and IgG seroconversion during convalescence. She fulfilled the case definition of RMSF. On the ninth day of illness, when she developed swelling of her extremities, she matched the clinical criteria of Kawasaki disease: fever more than 5 days, oral mucositis, skin rash, bulbar conjunctivitis and swelling of the hands and feet. Her clinical diagnosis of RMSF was followed by the diagnosis of Kawasaki disease 4 days after initiation of antimicrobial therapy for RMSF and she showed clinical improvement during that interval. Tick bite, petechial rash, pancytopenia, seroconversion for RMSF and clinical improvement with doxycycline are the distinct features of RMSF rather than evolving or incomplete KD. During the second phase of the illness, the patient developed swelling of her extremities, and resolution of symptoms with IGIV and aspirin therapy which is characteristic of KD.

The close temporal occurrence of RMSF and KD in this patient suggests four possibilities: the association may be entirely coincidental; a nonspecific serologic response to RMSF in a patient with KD; RMSF leading to vasculitis mimicking KD; RMSF etiologically related to KD as a superantigen interacting with the host immune system. The theory of coincidental occurrence of RMSF and KD in this patient cannot be ruled out. It is possible that the patient presented with incomplete features of KD during early hospitalization and was undergoing various clinical phases of KD. Although a nonspecific antibody response can be seen in some of the patients with KD similar to certain infectious diseases such as Epstein–Barr Virus infection and collagen vascular disease, this patient had clinical features of RMSF distinct from KD and she had seroconversion for RMSF antibody. The patient lived in a part of the United States where RMSF is not endemic. In 1981, Headings and Santosham described a patient with KD with serologic evidence of recent RMSF, but that patient did not have the clinical evidence of RMSF [3].

Both KD and RMSF are associated with vasculitis involving small and medium sized blood vessels. In an experimental investigation by Walker and colleagues, infection of primary chick embryo monolayer cells showed dilatation of the rough endoplasmic reticulum and cellular necrosis of human endothelial cells [4,5]. Rickettsia-like bodies have been found in biopsy specimens from the skin and lymph nodes of patients with Kawasaki disease and these microbodies were isolated from the peripheral blood of a patient with Kawasaki disease [6,7]. Rickettsia-like bodies were found in the monocytes of splenic pericapsular tissue from a child with Kawasaki disease [8].

The clinical presentation of KD is very similar to a number of diseases caused by exotoxins acting as superantigens, such as toxic shock syndrome. The report by Rowley *et al.* of IgA plasma cell infiltration of vascular walls in children with KD suggests that KD is the result of the stimulation of an antigen-driven immune response with plasma cell involvement [9].

If one believes that this patient had vasculitis due to RMSF and that her clinical resolution occurred after the combination of doxycycline and IGIV, then the role of IGIV in some patients with RMSF needs further exploration. IGIV has been shown to be effective in other superantigen related conditions such as Toxic Shock Syndrome and autoimmune thrombocytopenic purpura refractive to conventional management. IGIV may have clinical benefits in vasculitic diseases such as RMSF by exerting its effect on the vascular endothelium. There have not been any published reports of the use of IGIV in patients with RMSF.

We also did not find any literature documenting KD following clinical RMSF with seroconversion. Our patient, the first to be published, more likely represents KD following RMSF rather than a RMSF-related vasculitis. The patient had biphasic illness with two clearly separate symptom complexes. RMSF responded quickly to doxycycline and the KD responded quickly to IGIV.

## Conclusions

This case report suggests that Kawasaki disease can occur concurrently or immediately after a rickettsial disease such as RMSF. This led us to hypothesize that the etiologic agent of RMSF could act as a superantigen leading to immune-stimulation resulting in vasculitis seen in KD. In some patients with RMSF who have severe vasculitis and who are refractory to antimicrobial therapy, IGIV may have potential benefits, and needs to be studied further.

## Abbreviations

ELISA: enzyme-linked immunosorbent assay; IFA: immunofluorescence antibody; IGIV: immune globulin intravenous; IgM: Immunoglobulin M; IgG: Immunoglobulin G; KD: Kawasaki disease; RMSF: Rocky Mountain spotted fever.

## Consent

Written informed consent was obtained from the patient's mother for publication of this case report and any accompanying images. A copy of the written consent is available for review by the Editor in Chief of this journal.

## Competing interests

The authors declare that they have no competing interests.

## Authors' contributions

AB and SK were responsible for diagnosis, patient management, literature review and writing. Both authors read and approved the final manuscript.

## References

[B1] Taubert KA Epidemiology of Kawasaki disease in the United States and worldwide Prog Pediatric Cardiol 1997 6 181 185 10.1016/S1058-9813(97)00188-4

[B2] Burgner D Harnden A Kawasaki Disease: what is the epidemiology telling us about the etiology? Int J Infect Dis 2005 9 185 194 10.1016/j.ijid.2005.03.002 15936970 PMC7110839

[B3] Headings DL Santosham M Kawasaki disease associated with serologic evidence of Rocky Mountain Spotted Fever Johns Hopkins Med J 1981 149 220 221 7311262

[B4] Walker DH Cain BG The rickettsial plaque: Evidence for direct cytopathic effect of Rickettsia rickettsii Lab Invest 1980 43 388 396 6777602

[B5] Walker DH Firth WT Edgell CJ Human endothelial cell culture plaques induced by Rickettsia ricketsii Infect Immun 1982 37 301 306 6809631 10.1128/iai.37.1.301-306.1982 PMC347527

[B6] Tasaka K Hamashima Y Studies on rickettsia-like body in Kawasaki disease. Attempts of the isolation and characterization Acta Pathol Jpn 1978 28 235 245 354323 10.1111/j.1440-1827.1978.tb00535.x

[B7] Hamashima Y Kishi K Tasaka K Rickettsia-like bodies in infantile acute febrile lymph-node syndrome Lancet 1973 2 42 10.1016/S0140-6736(73)91975-2 4123314

[B8] Carter RF Haynes ME Morton J Rickettsia-like bodies and splenitis in Kawasaki disease Lancet 1976 2 1254 1255 10.1016/S0140-6736(76)91192-2 63083

[B9] Rowley AH Eckerley CA Jack HM Shulman ST Baker SC IgA plasma cells in vascular tissue of patients with Kawasaki syndrome J Immunol 1997 159 5946 5955 9550392

